# The Paraoxonase (*PON*) Gene Family in Health, Metabolic Dysfunction-Associated Steatotic Liver Disease (MASLD) and Other Diseases

**DOI:** 10.3390/ijms262211054

**Published:** 2025-11-15

**Authors:** Tammy Huybrechts, Kristien Franck, Ellen Steenackers, Wim Van Hul

**Affiliations:** Department of Medical Genetics, University of Antwerp, 2000 Antwerp, Belgium; tammy.huybrechts@uantwerpen.be (T.H.); kristien.franck@hotmail.com (K.F.); ellen.steenackers@uantwerpen.be (E.S.)

**Keywords:** paraoxonases, metabolic dysfunction-associated steatotic liver disease (MASLD), antioxidants

## Abstract

The Paraoxonase (*PON*) gene family consists of three paralogues (*PON1*, *PON2* and *PON3*) that are tandemly located on chromosome 7. In this review paper, the structure and function of the encoded proteins is summarized. In addition, an overview is given on the generated animal models. Finally, their involvement in the pathogenesis of different diseases is discussed, starting from an extended screening of the literature using PUBMED and Web of Science. PON1 and PON3 are mainly expressed in the liver and released into the bloodstream, bound to high-density lipoprotein. PON2 is expressed in various tissues, including the liver, lungs, heart, placenta and testes, but remains intracellular. The name of the enzyme family reflects PON1′s ability to neutralize paraoxon, but they also exhibit lactonase and esterase activities. All three PON enzymes play a role in reducing lipid peroxides in High-Density Lipoproteïne (HDL) and low-density lipoprotein(LDL), giving them antioxidant properties. This links them to Metabolic dysfunction-Associated Steatotic Liver Disease (MASLD), a metabolic liver condition marked by the excessive accumulation of triglycerides (TG) in liver cells. In addition to their association with MASLD, the *PON* genes are, due to their antioxidant properties, also associated with other conditions including cardiovascular diseases, chronic kidney disease, neurological and immunological conditions up to some forms of cancer. In the latter, the antioxidant properties can result in tumor progression by protecting malignant cells from oxidative damage thus supporting survival, proliferation and metastasis indicating them as potential drug targets for treatment of cancer. Therefore, further research on this protein family can provide novel insights into their function and their potential therapeutic applicability.

## 1. Introduction

Metabolic dysfunction-associated steatotic liver disease (MASLD), formerly known as non-alcoholic fatty liver disease (NAFLD), is a metabolic liver condition marked by the excessive accumulation of triglycerides (TG) in liver cells [1,2]. It has emerged as the most common chronic liver disorder worldwide. Recent estimates indicate that its prevalence in the general population has increased from approximately 25% in 2016 to over 30% currently. This rise is largely attributed to the increasing prevalence of obesity, type 2 diabetes mellitus (T2DM) and other (cardio)metabolic risk factors [3].

**Figure 1:**
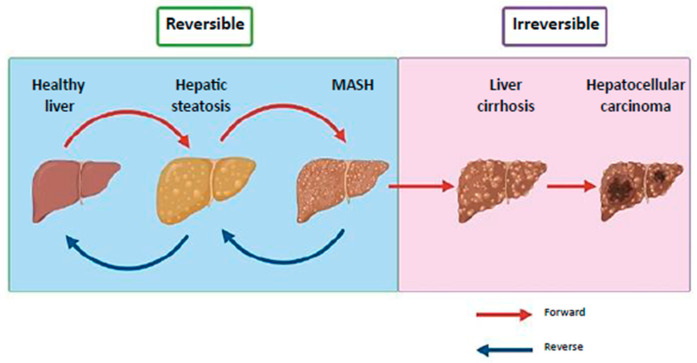
Clinical progression of MASLD. MASLD progresses from reversible hepatic steatosis and MASH to irreversible cirrhosis and hepatocellular carcinoma. Steatosis, marked by excess fat, can develop into MASH with inflammation and cellular damage, potentially leading to fibrosis and cirrhosis. MASLD, metabolic dysfunction-associated steatotic liver disease; MASH, metabolic dysfunction-associated steatohepatitis (Image modified from Khairnar et al.) [6].

MASLD includes a spectrum of liver conditions, from hepatic steatosis, a milder and reversible form, to hepatocellular carcinoma (HCC) (Figure 1) [2,4,5,6]. Hepatic steatosis is characterized by fat accumulation exceeding 5% of liver weight and may progress to metabolic dysfunction-associated steatohepatitis (MASH), which involves steatosis, inflammation and cellular injury [7]. MASH can further advance to liver fibrosis, cirrhosis and eventually HCC [4,8]. The two initial stages of MASLD are reversible, while the presence of cirrhosis is persistent (Figure 1) [4]. The onset and progression of MASLD is influenced by a combination of metabolic dysfunction, genetic predisposition and environmental factors [9]. In 2024, Resmetirom, a selective thyroid hormone receptor beta (THR-β) agonist, became the first U.S. Food and Drug Administration (FDA) approved drug that targets multiple stages of the disease. It is approved for adult patients with noncirrhotic MASH, where clinical trials revealed improvements in hepatic fat, liver fibrosis and positive effects on metabolic health and quality of life [10].

### The Paraoxonase Gene Family

An extended set of genes and proteins have been suggested to be involved in the pathogenesis of MASLD. These include the Paraoxonase (*PON*) gene family that consists of three members: *PON1*, *PON2* and *PON3*. In humans, the genes are tandemly located on chromosome 7 and exhibit significant structural similarity. In mice they are situated on chromosome 6, while in zebrafish they are located on chromosome 16 [11,12]. *PON2* is considered the oldest member of the family, with *PON1* and *PON3* likely arising from gene duplication events [12]. *PON1* and *PON3* are mainly expressed in the liver and released into the bloodstream, where they are bound to high-density lipoprotein (HDL) [13]. *PON3* is also expressed in the kidney [14]. Conversely, *PON2* is expressed in various tissues, including the liver, lungs, heart, placenta and testes, but remains intracellular and is not found in the circulation [15].

The enzyme family is named after paraoxonase, reflecting PON1′s ability to neutralize paraoxon, a toxic metabolite of the insecticide parathion [13]. This unique activity is not shared by PON2 or PON3, which do not degrade insecticides [16]. In addition to paraoxonase activity, PON1 also exhibits lactonase and other esterase activities [17]. All three PON enzymes play a role in reducing lipid peroxides in HDL and LDL, giving them antioxidant properties [16]. PON1, the most extensively studied of the group, has been linked to various oxidative stress-related conditions, including MASLD. Alterations in circulating PON1 levels have been observed in these diseases [18,19,20].

For this review, a literature search was performed in PubMed and Web of Science to identify relevant studies on the *PON* genes. The search included publications up to August 2025 and combined controlled vocabulary terms and free-text keywords. Search strings included combinations of “PON”, “paraoxonase”, “PON1”, “PON2”, and “PON3” together with terms such as “animal models”, “cancer”, “Atherosclerosis”, and “MASLD”. Additional relevant articles were identified by screening the reference lists of key papers and reviews. Only peer-reviewed articles published in English were included.

## 2. Paraoxonase-1

### 2.1. Structure of PON1

**Figure 2:**
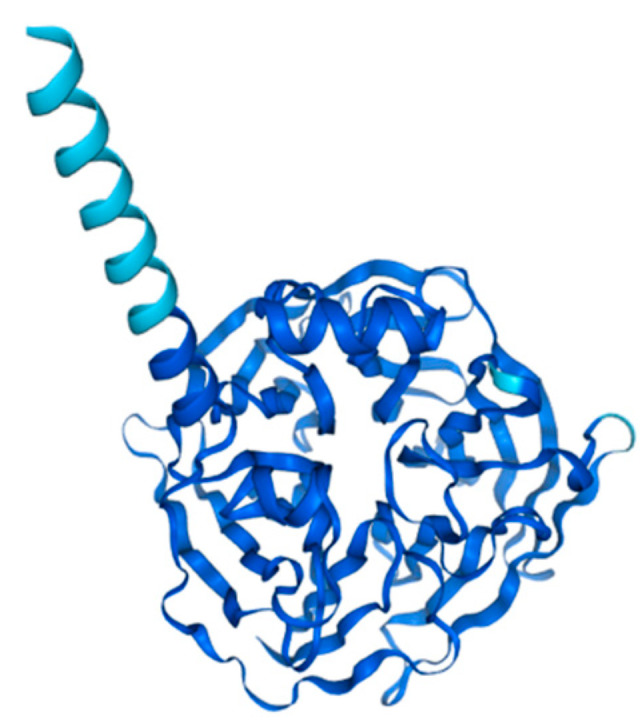
Structure prediction of PON1-201 from Alphafold v2.3.2. (ENSG00000005421-PON1 from the Human Protein Atlas.

PON1 is a hydrolytic enzyme that depends on calcium for its function. It is composed of 354 amino acids and has a molecular weight of 43 kDa. The enzyme features a six-bladed β-propeller structure, with each blade formed by four β-sheets. Within its core lies a central tunnel containing two calcium ions, both of which are essential for PON1′s functionality (Figure 2) [21,22,23]. One calcium ion serves a structural element, maintaining the enzyme’s conformational stability, while the other has a catalytic role, aiding in substrate alignment and the activation of ester bonds. Positioned above PON1′s active site are three helices, named H1, H2 and H3. The H1 and H2 helices are particularly important for facilitating interactions between PON1 and HDL [21,24].

### 2.2. Function of PON1: An Antioxidant Enzyme

**Figure 3:**
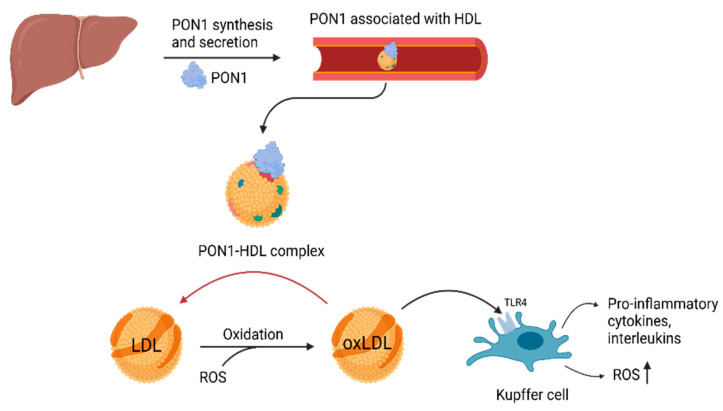
Antioxidant function of PON1. PON1, paraoxonase-1; HDL, high-density lipoprotein; LDL, low-density lipoprotein; oxLDL, oxidized low-density lipoprotein; ROS, reactive oxygen species; TLR4, Toll-like receptor 4 (Created with BioRender.com).

PON1 is a multifunctional enzyme that exhibits multiple enzymatic activities, including paraoxonase (hydrolysis of paraoxon), arylesterase (hydrolysis of aromatic esters) and lactonase (hydrolysis of lactones) [25,26,27]. Its lactonase function gives the enzyme its antioxidant and anti-inflammatory properties by breaking down lipid peroxides present in lipoproteins such as LDL [28,29]. Oxidative stress, a central factor in the development of MASLD, leads to the oxidation of LDL (oxLDL) through the action of reactive oxygen species (ROS) [20,30]. Oxidized LDL triggers Toll-like receptor signaling pathways, particularly TLR4 in Kupffer Cells (KCs), which results in the release of pro-inflammatory cytokines, interleukins and additional ROS [31,32]. PON1, which is associated with HDL in the bloodstream, hydrolyses oxLDL, converting it back into LDL (Figure 3) [30]. Through this mechanism, PON1 serves as a protective agent, mitigating oxidative stress and inflammation [30,33].

### 2.3. PON1 and MASLD: Reduced PON1 Activity

Reduced serum PON1 activity is observed in conditions characterized by oxidative stress and inflammation, such as MASLD [13,34]. Various mechanisms contribute to this reduction in activity, including the influence of pro-inflammatory cytokines. Individuals with MASLD or MASH exhibit elevated plasma levels of interleukin-6 (IL-6) and Tumor Necrosis Factor-alpha (TNF-α), which may interfere with PON1 serum levels [19]. Oxidized LDL contains reactive aldehydes that can covalently modify the sulfhydryl (-SH) group on cysteine-284 of PON1, impairing its function. These aldehydes, along with ROS, can oxidize the sulfhydryl to sulfenic acid (-SOH), which is reversible. However, excessive oxidative stress can convert it to irreversible forms like sulfinic (-SO_2_H) or sulfonic acid (-SO_3_H), permanently inactivating PON1. While PON1 can resist minor oxidative changes, sustained oxidative stress exceeds its capacity, reducing its ability to protect against further LDL oxidation [35,36,37]. As a result, under conditions of sustained oxidative stress, PON1 is inactivated by the excessive presence of its own substrate oxLDL, losing its antioxidant properties. Additionally, oxidative stress can inactivate PON1 through S-glutathionylation, a redox-regulatory process involving the formation of a mixed disulfide between cysteine-284 and oxidized glutathione [38]. Furthermore, oxidative stress has been associated with ER stress, disrupting proper protein folding and thereby impairing the secretion of functional proteins [39]. MASLD is also commonly linked to reduced HDL levels, reducing the availability of HDL for PON1 to bind [40]. Finally, genetic variations, such as the L55M and Q192R polymorphisms in the *PON1* gene, influence both the activity and concentration of PON1 [19].

### 2.4. PON1 and Other Diseases

#### 2.4.1. Role in Cardiovascular and Metabolic Diseases

PON1 is primarily associated with HDL in circulation, where it plays a key role in preventing the oxidative modification of LDL into oxLDL, a central driver of atherosclerotic plaque formation. Its antioxidant activity not only limits lipid peroxidation but also reduces vascular inflammation, further protecting against atherosclerosis [41]. Moreover, the association of PON1 with HDL enhances HDL functionality, thereby preserving its anti-atherogenic properties. This includes the inhibition of matrix metalloproteinase-9 (MMP-9)-mediated vascular remodeling, a process linked to endothelial dysfunction and plaque progression [42].

#### 2.4.2. Role in Chronic Kidney Disease (CKD) and Cardiac Injury in CKD

Chronic kidney disease (CKD) is a progressive inflammatory condition strongly associated with cardiovascular morbidity and mortality. Oxidative stress and inflammation play a central role in CKD progression, with cardiotonic steroids (CTS) identified as key contributors to renal inflammation and fibrosis via Na^+^/K^+^-ATPase signaling. Clinical studies consistently show reduced PON1 activity in CKD patients, which correlates with increased oxidative stress markers, low thiol levels, elevated CRP, and worse cardiovascular outcomes [43]. Dube et al. investigated the impact of PON1 deficiency on cardiac injury and dysfunction in a PON1 knockout rat model of salt-induced renal disease. In this salt-sensitive hypertensive setting, loss of PON1 resulted in compensated concentric left ventricular hypertrophy, characterized by decreased left ventricle (LV) chamber dimensions, increased relative wall thickness, and enhanced global systolic function. These findings were consistently supported by multiple echocardiographic measures, including fractional shortening, mean velocity of circumferential fiber shortening, and cardiac index, all indicating elevated systolic performance in the absence of PON1 [44].

##### Association with Immune Dysregulation

PON1 exerts anti-inflammatory effects through multiple mechanisms. It can suppress pro-inflammatory responses in macrophages by inhibiting the production and secretion of cytokines such as TNF-α and IL-6, a process mediated through interaction with the scavenger receptor class B type I (SR-BI) [45]. Additionally, PON1 influences adaptive immunity; CD4^+^ T cells from PON1-deficient models exhibit increased IFN-γ production, indicating a role for PON1 in modulating T cell-mediated inflammatory responses [46]. Bai et al. developed PON1 knockout rats to explore the role of PON1 in immune system regulation. PON1 deficiency resulted in significantly reduced T cell numbers in peripheral blood, spleen, and thymus. Mechanistically, this reduction was linked to a developmental block in thymocyte maturation at the transition from the CD4^−^CD8^−^ double-negative (DN) stage to the CD4^+^CD8^+^ double-positive (DP) stage. This arrest was associated with increased p38 phosphorylation in both DN and DP thymocytes lacking PON1, suggesting a role for PON1 in T cell development [47].

##### Association with Autoimmune and Inflammatory Diseases

Systemic lupus erythematosus (SLE) is a chronic inflammatory autoimmune disease that is strongly associated with an increased risk of coronary artery disease. Tripi et al. demonstrated a potential role for PON1 in SLE, showing that reduced PON1 activity towards paraoxon is linked to the disease [48]. A similar association has been observed in rheumatoid arthritis (RA), another chronic inflammatory condition. In a study by Baskol et al., PON1 antioxidant activity was significantly reduced in RA patients, while levels of the oxidative stress marker malondialdehyde (MDA) were markedly elevated. These findings support the hypothesis that increased levels of reactive oxygen species (ROS) in RA create a pro-oxidative environment, leading to reduced PON1 activity and elevated lipid peroxidation, as reflected by higher MDA concentrations [49].

#### 2.4.3. Role in Neurological Diseases

A role for PON1 has also been identified in neurological disorders, particularly in Alzheimer’s disease and Parkinson’s disease (PD). Reduced PON1 activity is associated with increased oxidative stress and elevated levels of lipid peroxidation products, both of which are key contributors to the pathogenesis of late-onset Alzheimer’s disease and vascular dementia [50]. Erlich et al. further demonstrated that specific *PON1* polymorphisms are linked to an increased risk of Alzheimer’s disease, reinforcing the enzyme’s involvement in neurodegeneration [51]. Additionally, polymorphisms at positions 55 and 192 have been associated with decreased PON1 activity and serum levels in patients with PD [52].

#### 2.4.4. Role in Cancer

The role of PON1 in dealing with oxidative stress, inflammation, and detoxification makes it biologically plausible that variation in PON1 could affect cancer risk [53]. For example, in lung cancer, experimental work suggests that PON1 overexpression in certain contexts can actually aid tumor progression by protecting cells from oxidative damage and supporting survival, proliferation and metastasis [54]. The PON1 L55M variant (rs854560) appears to be associated with overall cancer risk, particularly in breast cancer and hematological cancers [55]. The PON1 Q192R variant (rs662) is more variable in findings. Some meta-analyses find associations (e.g., for breast cancer) while others find little overall effect [56]. In addition, there are reports that PON1 enzyme activity is lower in cancer patients compared to healthy controls in various cancer types (lung, breast, gastrointestinal, etc.), which might reflect a reduced antioxidant defense [57].

Together these data indicate that PON1, oxidative stress, chronic inflammation, and cancer can be closely linked. 

### 2.5. Animal Models

Research utilizing PON1 knockout (Pon1^−/−^) animal models has underscored the critical protective functions of PON1 in various pathological conditions. An overview of these models is provided in Table 1. Depending on the specific disease model, animals were exposed to standard chow, atherogenic diets, high-fat Western diets, or high-salt diets to investigate the role of PON1 in disease onset and progression. PON1-deficient mice exhibited markedly increased sensitivity to organophosphate compounds, highlighting the enzyme’s essential role in their detoxification [41]. In addition, these models demonstrated elevated levels of oxLDL and enhanced oxidative stress in macrophages, contributing to the development of atherosclerosis [58]. In high-salt rat models, the absence of PON1 led to renal injury, as well as cardiac and renal fibrosis and inflammation [43,44]. Collectively, these findings emphasize the multifaceted protective role of PON1 in limiting oxidative damage, modulating inflammation, and facilitating the detoxification of harmful organic compounds.

### 2.6. PON1 Polymorphisms

Human *PON1* exhibits numerous SNPs that can influence both the likelihood of developing diseases and their severity [77,78]. Research has identified two prevalent polymorphisms in the coding region that impact PON1 activity and concentration [79]. One such polymorphism, involving a leucine to methionine change at position 55 (L55M, rs854560), is linked to variations in PON1 serum concentrations [80]. A study by Milaciu et al. found that individuals with heterozygous (LM) or homozygous (MM) forms of the L55M polymorphism tend to have reduced PON1 concentrations. Additionally, the M allele of this polymorphism was observed more frequently in patients with MASLD [19]. Another significant polymorphism, involving glutamine or arginine at position 192 (Q192R, rs662), affects enzyme activity. The R192 variant has been shown to more effectively hydrolyze paraoxon and metabolize oxidized LDL compared to the Q192 variant [81]. Furthermore, a polymorphism in the promoter region of the gene, C-108T (rs705379), plays a key role in regulating *PON1* expression. Individuals with the C allele tend to exhibit higher serum levels of PON1 [27].

Finally, an association between rare and very rare PON1 variants and obesity was reported [28].

## 3. Paraoxonase-2

### 3.1. Structure of PON2

**Figure 4:**
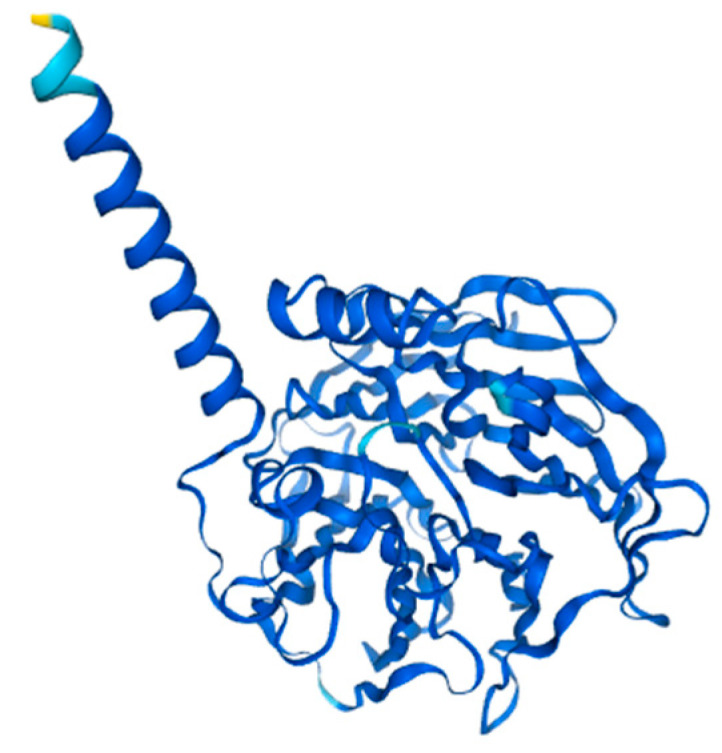
Structure prediction of PON2-201 from Alphafold v2.3.2. (ENSG00000105854-PON2) from the Human Protein Atlas.

*PON2* is the oldest member of the Paraoxonase gene family from which *PON1* and *PON3* evolved later [82]. *PON2* is a widely expressed intracellular enzyme with the highest concentrations in the perinuclear region, endoplasmic reticulum, and mitochondria. The enzyme is composed of 355 amino acids, arranged into 9 exons, and has a molecular weight ranging from 40 to 43 kDa (Figure 4) [83]. Several transcription start sites have been observed in the *PON2* sequence but only the 40 kDa and 43 kDa isoforms have been observed with immunoblotting with a specific anti-PON2 antibody [84].

All *PON* genes have diverged throughout evolution while retaining their hydrolytic machinery and active site. The structural model of PON2 has been developed based on existing knowledge of PON1, revealing shared protein segments such as histidine and cysteine residues, as well as Ca^2+^-binding loops. Studies have confirmed that the highly conserved glycosylation sites of PON1 (Asn253 and Asn324), which are essential for its structural integrity and high catalytic activity, are also present in PON2 (Asn254 and Asn323), where they similarly play a key role in modulating its lactonase activity [85,86]. PON2 is a type II transmembrane protein with a short N-terminal domain located in the cytoplasm, followed by a single α-helical transmembrane domain composed of highly hydrophobic residues (Figure 4). This domain functions similarly to the hydrophobic signal peptide that enables PON1 binding to HDL particles. The C-terminal region, which contains the active site (His114 and His133), is positioned extracellularly [87,88].

### 3.2. Function of PON2

PON2 has two important functions: an enzymatic hydrolytic activity and the reduction in oxidative stress, which is performed independently of the enzymatic activity. With its calcium-dependent hydrolytic activity, PON2 is able to hydrolyze lactones, esters and aryl esters. With its lactonase activity, it dominantly hydrolyses N-(3-oxo-dodecanoyl)-L-homoserine lactone (3OC12-HSL), a key quorum sensing signal of *P. aeruginosa.* PON2 thus plays an important role in innate immunity. PON2 exerts its antioxidant function primarily in the mitochondria and endoplasmic reticulum, where it is predominantly localized. With its active site facing the extracellular compartment, it protects membrane components from peroxidation.

In the mitochondria, PON2 helps to maintain coenzyme Q10 (CoQ10) in its reduced state, preventing its oxidation. CoQ10 is a crucial component of the electron transport chain (ETC), where it functions as an electron carrier between complex I and complex III, as well as between complex II and complex III. By reducing oxidative stress and stabilizing CoQ10, PON2 helps to limit the formation of ROS, which can arise from the unstable intermediate ubisemiquinone (*CoQH^•^*) [84,86,89].

In the endoplasmic reticulum (ER), PON2 expression is upregulated in response to ER stress through increased promoter activity [90]. This upregulation plays a crucial role in inhibiting apoptosis. PON2 reduces caspase activation mediated by the unfolded protein response (UPR) [84]. Additionally, PON2 suppresses apoptosis by downregulating the pro-apoptotic transcription factor CHOP (CEBP homologous protein). Upon ER stress, PON2 overexpression leads to reduced CHOP levels, thereby promoting cell survival [91].

### 3.3. PON2 and MASLD

Recent studies by Shin et al. have highlighted a role for PON2 in various stages of MASLD. Loss of PON2 has been linked to several key pathways involved in MASLD pathogenesis, including mitochondrial respiratory capacity, lipid metabolism, hepatic fibrosis, and inflammation.

Using an in vitro hepatocyte model (L02 cells) with stable PON2 knockdown (KD), cells were treated with palmitic acid (PA) to mimic the effects of a Western diet. PON2-deficient cells exhibited greater lipid droplet accumulation following PA treatment, alongside slower lipolysis compared to control cells. Additionally, genes involved in fatty acid metabolism were downregulated in PON2-deficient cells, whereas genes related to cholesterol and ceramide metabolism were upregulated. These findings suggest that PON2 plays a crucial role in lipid homeostasis by regulating key genes involved in lipid metabolism.

Mitochondrial function was also impaired in PON2-depleted cells, as evidenced by reduced mitochondrial respiration and increased mitochondrial superoxide production, with these effects being exacerbated after PA treatment. Furthermore, genes involved in the electron transport chain (ETC) and the Tricarboxylic Acid (TCA) cycle were significantly downregulated in PON2-deficient cells, indicating that PON2 is essential for maintaining mitochondrial metabolic activity and protecting against oxidative stress-induced mitochondrial damage.

To investigate PON2′s role in inflammation, levels of malondialdehyde (MDA)—a marker of lipid peroxidation—were measured in both control and PON2-depleted cells, with and without PA treatment. PON2-deficient cells showed higher MDA levels, both under basal conditions and following PA exposure. Gene expression analysis further revealed that PON2 depletion upregulated pathways involved in macrophage chemotaxis, migration, inflammation, and steatohepatitis, while genes related to platelet inactivation were downregulated. These findings suggest that loss of PON2 exacerbates inflammation and promotes the accumulation of oxidized lipid metabolites in hepatocytes.

Finally, given the strong link between autophagy, mitochondrial dysfunction, and oxidative stress, the role of PON2 in autophagy was investigated. Analysis of autophagy-related markers revealed that PON2 depletion reduced autophagosome formation and autophagic flux, suggesting that PON2 is essential for maintaining autophagic activity.

These findings indicate that PON2 is a key regulator of lipid metabolism, mitochondrial function, oxidative stress, and inflammation in hepatocytes. Its depletion worsens lipid accumulation, impairs mitochondrial respiration, increases oxidative stress, disrupts autophagy, and exacerbates inflammation, highlighting its potential role in MASLD progression [92].

Later, the same researchers identified PON2 as a promising therapeutic target for slowing MASLD progression. They synthesized vutiglabridin (VUTI), a derivative of glabridin, which offers potential therapeutic benefits for MASLD due to its enhanced chemical stability and improved oral bioavailability. Unlike glabridin, previously explored as a treatment for metabolic diseases, vutiglabridin presents a more viable option for clinical application [93,94]. The mechanisms of action and cellular targets of VUTI were investigated using the L02 PON2 KD cell line, primary human hepatocyte spheroids, and mouse models. In vitro, VUTI significantly improved lipid accumulation in both hepatocyte spheroids and the cell model. It also influenced multiple mitochondrial processes, enhancing ATP production and maximal respiratory capacity while reducing PA-induced mitochondrial superoxide levels and lipid peroxidation. Additionally, VUTI promoted mitophagy activation, suggesting its role in maintaining mitochondrial quality control.

To determine VUTI’s cellular localization, a fluorescence-tagged version was used, revealing that VUTI primarily localizes to the mitochondria. A pull-down assay using biotin-labeled VUTI and cell lysates identified 130 mitochondrial proteins as potential binding partners. Gene ontology analysis of these proteins showed enrichment in pathways related to oxidative phosphorylation, fatty acid oxidation, the TCA cycle, and oxidative stress homeostasis, all of which are crucial for mitochondrial energy metabolism. PON2 emerged as the primary target of VUTI, as it is highly expressed in the mitochondria and plays a key role in maintaining mitochondrial function. This was further validated through a competitive pull-down assay, confirming direct binding of VUTI to PON2.

Previous research had already demonstrated that lipotoxic conditions inhibit PON2 activity, and VUTI was found to restore PON2 activity under these conditions without altering PON2 expression levels. Importantly, the beneficial effects of VUTI on mitochondrial function were absent in PON2 KD cells, reinforcing the notion that PON2 is essential for VUTI’s mitochondrial protective effects.

To assess its therapeutic potential, VUTI was tested in an amylin diet-induced obese (AMLN-DIO) Non-Alcoholic SteatoHepatitis (NASH) mouse model, which exhibits severe liver steatosis and inflammation. Treatment with VUTI alleviated steatosis, liver fibrosis, and intrahepatic monocyte infiltration, while biochemical assays showed that VUTI reduced serum and hepatic cholesterol levels. Immunostaining analyses further confirmed that VUTI promoted autophagy activation in the liver, supporting the observations from in vitro models.

Overall, these findings highlight VUTI’s potential as a therapeutic agent for MASLD, primarily through its mitochondrial protective effects via PON2 activation. By restoring PON2 activity, VUTI improves lipid metabolism, reduces oxidative stress, and enhances mitochondrial function, making it a promising candidate for further investigation in metabolic liver diseases [93].

These findings reinforce the importance of the *PON* gene family in the development and progression of MASLD, further highlighting their relevance as potential therapeutic targets [28].

### 3.4. PON2 and Other Diseases

Besides its role in MASLD, PON2 research revealed a broad range of diseases in which PON2 plays a role.

#### 3.4.1. Role in Cardiovascular Disease and Atherosclerosis

A common PON2 polymorphism (Cys311Ser) has been associated with an increased risk of cardiovascular disease in individuals with familial hypercholesterolemia, suggesting that genetic variation in PON2 may influence individual susceptibility to atherosclerosis [95]. Atherosclerosis is believed to develop primarily through the oxidative modification of LDL, a key driver of vascular inflammation and plaque formation. PON2 plays a protective role by inhibiting LDL oxidation and reducing cellular oxidative stress and ROS [96]. Experimental studies in PON2 knockout (Pon2^−^/^−^) mice further support its role in the initiation and progression of atherosclerosis, as summarized in Table 1. Moreover, a recent meta-analysis in patients with coronary heart disease (CHD) identified a protective association of the Ser311Cys variant, particularly in the overall and Asian populations, reinforcing the clinical relevance of PON2 genetic variation in cardiovascular risk.

#### 3.4.2. Role in Neurodegenerative Diseases

PON2 has emerged as an important modulator of neurodegenerative disease through its antioxidant and anti-inflammatory functions. In Alzheimer’s disease, *PON2* expression and polymorphisms, particularly the C311S variant, have been associated with increased susceptibility, likely by altering resistance to oxidative stress [97]. In PD, PON2 interacts with the PD-related protein DJ-1 to enhance neuronal survival under oxidative conditions [98]. The neurotoxin 1-methyl-4-phenylpyridinium ion (MPP+) is a widely used neurotoxin to induce PD in animal models. β-estradiol-3-benzoate (EB) exerts neuroprotective effects in MPP+ induced experimental models of PD by upregulating PON2 expression in the striatum [99]. Recently, several papers described an association between PON2 C311S and amyotrophic lateral sclerosis (ALS) [99,100].

#### 3.4.3. Role in Cancer

Multiple studies have demonstrated that PON2 contributes to tumor growth and metastatic progression. Elevated PON2 expression has been associated with enhanced tumor aggressiveness in several malignancies, including colorectal cancer, skin cancer and lung adenocarcinoma. By supporting cellular proliferation, survival, and therapy resistance, PON2 acts as a critical regulator of cancer cell fitness, effects that are markedly diminished upon PON2 knockdown [71,101,102,103,104,105,106]. *PON2* overexpression protects cancer cells from ROS-induced apoptotic cell death. Conversely, silencing PON2 reverses this effect by enhancing ROS accumulation. Similarly, specific mutations in PON2, as observed in lung cancer cells, impairs its antioxidative function [86,101,107].

Through its regulation of ROS and oxidative stress, PON2 is a key determinant of cancer cell proliferation, survival, and treatment response across multiple tumor types [108]. All these data suggest that PON2 represents a promising therapeutic target for treatment of some types of cancer. This was recently nicely illustrated by Belloni et al. for Cisplatin, a chemotherapy drug used for several cancers, that showed an increased sensitivity of cancer cells after PON2 knockdown [109].

### 3.5. Animal Models

Studies using PON2-deficient (*Pon2^−^/^−^*) mouse models have revealed a broad protective role for paraoxonase-2 (PON2) across multiple organ systems. These effects are primarily mediated through its modulation of oxidative stress, mitochondrial homeostasis, inflammation, and lipid metabolism. An overview of the experimental PON2 models and their phenotypic outcomes is provided in Table 1.

Under standard chow diet conditions, PON2 expression was found to be especially enriched in regions of the brain with high oxidative metabolism, such as the hippocampus and substantia nigra, supporting its role in counteracting local oxidative stress [63,72]. PON2-deficient mice exhibited increased markers of oxidative stress in these brain regions, as well as impaired mitochondrial function in retinal pigment epithelial cells [72]. In the cardiovascular system, loss of PON2 led to elevated IL-6 levels, increased tissue factor activity, and vascular endothelial dysfunction [65]. Additionally, PON2 deficiency exacerbated myocardial damage following ischemia–reperfusion injury, highlighting its role in cardio protection [66].

Beyond oxidative regulation, PON2 was shown to influence hematopoietic stem cell (HSC) function. *Pon2^−^/^−^* mice displayed skewed differentiation of HSCs, associated with increased ROS levels [69]. Furthermore, PON2 has been implicated in tumor biology, but further in vivo research is necessary to confirm in vitro results [71].

Under atherogenic dietary conditions, such as cholesterol-rich or Western-type diets, PON2-deficient mice exhibited altered lipoprotein profiles with elevated Very Low-Density Lipoprotein (VLDL) and decreased HDL levels, alongside significantly enlarged atherosclerotic lesions [61]. These metabolic disturbances were further associated with impaired hepatic insulin signaling, reinforcing PON2′s role in protecting against diabetic vascular complications and insulin resistance [64].

When exposed to high-fat Western diets, *Pon2^−^/^−^* mice displayed increased endoplasmic reticulum (ER) stress, reduced mitochondrial efficiency, and higher expression of inflammatory markers in adipose and hepatic tissues. These observations confirm PON2′s function in maintaining metabolic homeostasis and limiting diet-induced obesity-related oxidative stress [67].

Together, these findings underscore the multifaceted role of PON2 in maintaining systemic homeostasis and preventing the onset or progression of various pathologies, including cardiovascular disease, metabolic dysfunction, neurodegeneration and cancer.

### 3.6. PON2 Polymorphisms

In 1996, two single nucleotide polymorphisms (SNPs) were detected in the *PON2* coding sequence: S311C (rs6954345) and A148G (rs11545941). These variants exhibit strong linkage disequilibrium, resulting in two commonly detected haplotypes: A148 with S311 and G148 with C311. The A148/S311 homozygous haplotype has been associated with elevated levels of plasma total cholesterol, LDL cholesterol, and Apolipoprotein B (apoB) [110,111].

Numerous studies have investigated the association of these SNPs with various diseases, but findings remain inconsistent due to differences in experimental design, ethnic backgrounds, and geographical locations. The S311C polymorphism has been associated with coronary artery disease (CAD), acute myocardial infarction (AMI), type 2 diabetes with ischemic stroke, glycemic control in type 2 diabetes, microalbuminuria in type 1 diabetes, Alzheimer’s disease, sporadic amyotrophic lateral sclerosis (ALS), familial hypercholesterolemia (FH), and altered levels of total cholesterol and LDL.

The A148G polymorphism has been linked to total cholesterol and LDL levels, fasting plasma glucose in type 2 diabetes, and low birth weight. However, further research is needed to validate these associations, as current findings remain contradictory [112].

Both variants have been linked to reduced PON2 activity, resulting in a significant decrease in both esterase and lactonase activity. This reduction is particularly pronounced for 3OC12-HSL, which is believed to be the primary biological substrate of PON2. [85,113].

Recently variant screening in patients without fibrosis or with varying fibrosis stages, indicated highly significant associations for rare and very rare PON2 variants, suggesting a potential role for PON2 in fibrosis progression. These findings reinforce the importance of the PON gene family in the development and progression of MASLD, further highlighting their relevance as potential therapeutic targets [28].

## 4. Paraoxonase-3

PON3 is the least characterized member of the paraoxonase enzyme family. It was the last to be identified and remains the most enigmatic in terms of its biological function [114]. PON3 is a 40 kDa glycoprotein primarily synthesized in the liver, with lower levels of expression in the kidney [16]. In circulation, it is associated with HDL, albeit at significantly lower concentrations than PON1 [115].

### 4.1. Structure of PON3

**Figure 5:**
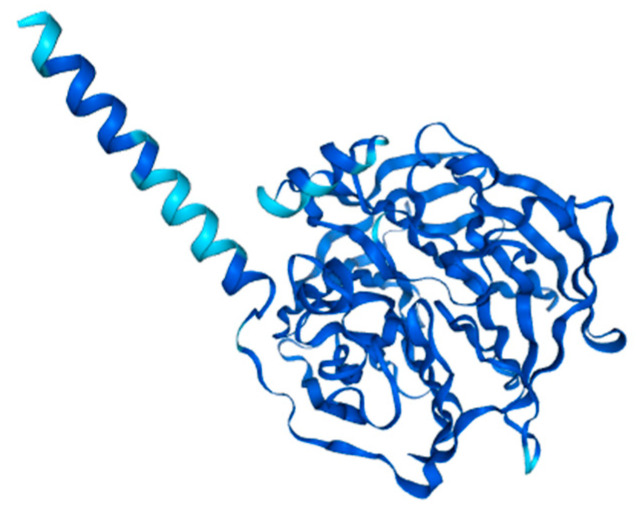
Structure prediction of PON3-201 from Alphafold v2.3.2. (ENSG00000105852-PON3 from the Human Protein Atlas.

PON3 is a calcium-dependent enzyme, similar to PON1 (Figure 5) [116]. It exhibits lactonase activity, with a particularly high catalytic efficiency for hydrolyzing statin lactones such as lovastatin, which is commonly used to assess its activity [115,116,117]. Although its crystal structure remains undetermined, studies suggest that PON3 possesses a larger active site than PON1 and PON2, enabling it to hydrolyze bulkier substrates [114,115,117].

### 4.2. Function of PON3

PON3, like PON1 and PON2, possesses antioxidant properties. Draganov et al. have reported that rabbit PON3 purified from serum is capable of inhibiting copper-induced LDL oxidation in vitro, highlighting its role in protecting against oxidative damage [115]. Interestingly, PON3 appears to be more potent than PON1 in protecting LDL from oxidative modification in vitro, although PON3 concentration in serum is about two orders of magnitude lower than PON1 [115]. Unlike PON1, liver PON3 expression is not affected by oxidized phospholipids (HepG2 cells) or a high-fat diet (mice liver) [16]. PON3 expression does not appear to change significantly in response to oxidative stress, whereas PON1 is downregulated and PON2 is upregulated [16,96].

Additionally, PON3 exhibits aryl esterase activity, although to a lesser extent than PON1 [115]. Despite the discovery of some potential biological substrates, the true physiological substrate for this enzyme remains unclear [114].

### 4.3. PON3 and MASLD

Given that PON3 is primarily synthesized in the liver and plays significant roles in lipid metabolism and oxidative stress regulation, it may be implicated in MASLD. PON3′s ability to protect against oxidative damage, enhance cholesterol efflux, and inhibit LDL oxidation suggests a potential protective role in MASLD progression.

Since PON3 remains stable under oxidative stress conditions and is not downregulated by a high-fat diet [16,96], it may contribute to mitigating the oxidative damage seen in MASLD. Furthermore, PON3′s hepatoprotective properties, demonstrated in models of chemically induced liver injury [118], indicate its potential role in reducing hepatic inflammation and fibrosis in MASLD.

### 4.4. Role in Human Health and Disease

#### 4.4.1. Role in Cardiovascular and Metabolic Diseases

PON3 appears to play a crucial role in cardiovascular health. In the study led by Ng et al., adenoviral expression of human PON3 protected apolipoprotein E knockout (apoE^−/−^) mice against progression of atherosclerosis. In particular, elevated levels of PON3 enhance cholesterol efflux, decrease LDL oxidation, and improve the antioxidant properties of HDL, all of which contribute to slowing the progression of atherosclerosis [119]. Studies using human PON3 transgenic mice have shown that increased PON3 activity is associated with a significant reduction in atherosclerotic lesion formation and adiposity [120].

A protective role of PON3 in obesity has been demonstrated in vivo in PON3 knockout (PON3^−/−^) mice. A lack of PON3 led to increased body weight, altered bile acid metabolism, and increased atherosclerotic lesions compared to WT mice on a high-fat diet. In addition, PON3 deficiency seemed to result in impaired mitochondrial respiration and mitochondrial superoxide levels and increased hepatic expression of inflammatory genes [74]. Additionally, an inverse correlation between PON3 concentration in HDL particles and body mass index was observed in patients with systemic lupus erythematosus, suggesting a role in adiposity regulation [121].

#### 4.4.2. Role in Immune-Mediated and Infectious Diseases

Additionally, PON3 contribute to defense mechanisms against *Pseudomonas aeruginosa* infections, primarily due to their antioxidative and anti-inflammatory properties. [122].

PON3 mitigates PCN-induced oxidative damage and exhibits anti-inflammatory properties by preventing NF-κB activation and reducing IL-8 secretion. However, *P. aeruginosa* has developed a countermeasure to evade these defenses. 3OC12 triggers intracellular calcium flux, leading to the rapid inactivation and degradation of both PON2 and PON3, highlighting a shared vulnerability that compromises their protective functions [122].

#### 4.4.3. Association with Autoimmune Diseases

PON3 concentrations have been found to be significantly elevated in chronic liver disease [123], HIV infection [124] and coronary and peripheral artery disease [125]. However, in patients with autoimmune diseases such as systemic lupus erythematosus and type 1 diabetes with subclinical atherosclerosis, PON3 levels in HDL are markedly reduced [121].

The measurement of PON3 in these studies has varied, with in-house serum ELISA assays used in earlier research [126], while later studies employed liquid chromatography tandem mass spectrometry (LC-MS/MS) and Western blot analysis to confirm findings [121].

#### 4.4.4. Protective Role in Liver Health

PON3 has hepatoprotective properties, preventing histological changes and liver cell apoptosis associated with liver disease [123]. Studies have shown that PON3 delivery to mice with carbon tetrachloride (CCl4)-induced liver injury significantly improved liver histological architecture, reinforcing its protective role [118]. This hepatoprotective effect of PON3 is closely related to its lactonase and antioxidant activities. PON3 expression was also significantly reduced in CCl4-treated rats, further linking it to liver injury prevention [127]. The protective role of PON3 in liver disease is expected since paraoxonases have a protective role against oxidative stress, which plays an important role in the pathogenesis of liver disease [128].

#### 4.4.5. Dual Role in Cancer

Despite its protective effects against oxidative stress-related diseases, PON3 has been found to exhibit oncogenic properties. It is overexpressed in several human cancers, where it protects tumor cells from mitochondrial superoxide-mediated apoptosis. This anti-apoptotic function may allow tumor cells to evade cell death, contributing to cancer progression [129].

Conversely, in HCC, PON3 appears to act as a tumor suppressor. Its downregulation has been associated with disease progression, suggesting that PON3 may serve as a prognostic marker in HCC [130,131].

### 4.5. PON3 Polymorphisms

Polymorphisms in the *PON3* gene have been identified in healthy individuals from southern Italy, but their functional consequences are yet to be evaluated. Two missense single nucleotide variants (SNVs), S311T (rs1053275) and G324D (rs13226149), have been detected, though at significantly lower frequencies compared to coding region variants of PON1 and PON2 [132].

### 4.6. Animal Models

Although less extensively studied than PON1 and PON2, PON3 has emerged as a key regulator of metabolic and renal homeostasis, as evidenced by findings from Pon3^−^/^−^ knockout animal models (Table 1). These models, generated in both mice and rats, were subjected to various dietary conditions, including standard chow, high-fat Western diets, cholesterol/cholate (CC) diets, and high-salt diets, to investigate the physiological role of PON3 across different disease contexts.

Under standard chow conditions, PON3 deficiency in mice led to early fetal/neonatal death and impaired fetal growth, highlighting its importance in embryonic development [73]. When challenged with a Western or CC diet, Pon3^−^/^−^ mice displayed increased susceptibility to obesity, altered lipid profiles, increased gallstone burden, and exacerbated atherosclerotic lesion development, supporting a role for PON3 in metabolic regulation and vascular protection [74]. In salt-loaded conditions, both Pon3^−^/^−^ rats and mice exhibited dysregulated renal function, including increased excretion of cardiotonic steroids and altered Na^+^ and K^+^ handling, suggesting that PON3 contributes to renal electrolyte balance and protects against salt-induced kidney injury [75,76].

## 5. Conclusions and Future Perspective

The paraoxonases are multifaceted enzymes with diverse biological functions, ranging from lipid metabolism and cardiovascular protection to roles in immunity, liver health, and cancer. While they exhibit strong antioxidant and anti-atherogenic properties, their oncogenic potential presents a paradox that requires further investigation, with already-available data indicating knockdown of the paraoxonases as a treatment option for different types of cancer. On the other hand, given their significant role in liver function and oxidative stress resistance, the paraoxonases may represent promising biomarkers and targets for therapeutic interventions in MASLD and other disorders.

## Figures and Tables

**Table 1:** Animal models for the *PON* genes.

**Author**	**Year**	**Pon1^−/−^ Model**	**Modification**	**Age**	**Diet**	**Investigation**	**Conclusion**
Shih, D. et al. [41]	1998	C57BL/6J mice	Neomycin cassette insertion in exon 1	Adult	Standard chow	Organophosphate toxicity	PON1-null mice showed dramatic sensitivity to chlorpyrifos oxon toxicity
					Atherogenic (high-fat, high-cholesterol; 15 weeks)	Atherosclerosis	PON1 deficiency led to impaired protection against LDL oxidation, causing significantly larger aortic atherosclerotic lesions compared to controls
Shih, D. et al. [59]	2000	Combined PON1 KO/ apoE KO mice (97% C57BL/6J 3% 129/SvJ)	Neomycin cassette insertion in exon 1	3 months	Standard chow (6% fat)	Atherosclerosis (increased lipoprotein oxidation and lesion formation)	PON1 deficiency resulted in higher levels of oxidized LDL and accelerates atherosclerosis in the pon1 KO/apoE KO mice
			Neomycin cassette insertion in exon 1	6 months	High-fat “Western” (42% fat, 0.15% cholesterol; 16 weeks)		
Rozenberg, O. et al. [58]	2003	C57BL/6J mice	Neomycin cassette insertion in exon 1	4 months	Standard chow	Increased macrophage oxidative stress and atherosclerosis	PON1 deficiency increases oxidative stress in macrophages, contributing to accelerated atherosclerosis
Givvimani, S. et al. [42]	2015	C57BL/6J mice	Purchased from Jackson Laboratory	8 weeks	Atherogenic (high-fat, high-cholesterol, cocoa butter; 15 weeks)	Endothelial dysfunction and atherosclerosis	Lack of PON1 leads to dysfunctional HDL, resulting in endothelial impairment and atherosclerosis via MMP-9–induced remodeling
Bai, L. et al. [47]	2018	Sprague-Dawley rats	342 bp deletion in exon 4 via CRISPR/Cas9	2 months	Standard chow	Impaired T cell development	PON1 deficiency leads to reduced thymocyte numbers and impaired T cell maturation, associated with increased apoptosis and upregulation of p38 MAPK signaling
Sikora, M. and Jakubowski, H. [60]	2021	C57BL/6J mice	Neomycin cassette insertion in exon 1	4 months	Standard chow vs. hyperhomocysteinemic (1% methionine in water)	Redox imbalance and atherogenic cardiovascular risk	Pon1 deficiency dysregulates redox proteins, explaining the pro-oxidant and pro-atherogenic phenotype
Khalaf, P. et al. [43]	2022	Dahl/SS rat	7-bp frameshift insertion in exon 4 via CRISPR/Cas9	10 weeks	High-salt (8% NaCl; 5 weeks)	CKD (renal inflammation, fibrosis, and hypertensive renal injury)	Loss of PON1 exacerbates renal injury, fibrosis, oxidative stress, and increases mortality, suggesting a protective role in CKD
Dube, P. et al. [44]	2022	Dahl/SS rat	7-bp frameshift insertion in exon 4 via CRISPR/Cas9	10 weeks	High-salt (8% NaCl; 5 weeks)	Cardiac injury in CKD	Loss of PON1 increases cardiac inflammation, fibrosis, and hypertrophy, indicating a cardioprotective role for PON1
**Author**	**Year**	**Pon2^−/−^ model**	**Modification**	**Age**	**Diet + Disease induction**	**Investigation**	**Conclusion**
Carey J. Ng et al. [61]	2006	C57Bl6/J mice	Insertional mutation in intron 2	Adult	Atherogenic diet (15.8% fat, 1.25% cholesterol, 0.5% cholic acid)	Atherosclerosis	PON2-deficient mice have lower serum levels of VLDL/LDL cholesterol and developed significantly larger atherosclerotic lesions.
Edna Meilin et al. [62]	2010	C57Bl6/J mice	Insertional mutation in intron 2	8–10 weeks	Standard chow	Macrophage triglycerides accumulation	PON2 has a protective role in reducing macrophage triglyceride accumulation, triglyceride biosynthesis, microsomal DGAT1 activity, and oxidative stress under high glucose conditions in cultured cells from knockout mice. This protective effect is likely mediated through the inhibition of NADPH-oxidase activity.
Giordano G. et al. [63]	2011	C57Bl6/J mice	Insertional mutation in intron 2	1–60 days	Standard chow	PON2 expression and activity in different mouse brain regions	PON2 has the highest expression levels in lung and small intestine, followed by heart and liver. Lower levels were found in testis, kidney and brain. Overall, a higher expression was observed in females. In the brain, PON2 protein levels were highest in nucleus accumbens, substantia nigra and striatum, areas known for high levels of oxidative stress. The pattern of PON2 lactonase activity followed the regional and gender profiles.
Noam Bourquard et al. [64]	2011	C57Bl6/J mice	Insertional mutation in intron 2	Adult	Standard chow, Atherogenic diet (15.8% fat, 1.25% cholesterol, 0.5% cholic acid) or Western diet (42% fat and 0.15 % cholesterol)	Hepatic insulin signaling	PON2 deficiency increases atherosclerosis susceptibility and is associated with impaired hepatic insulin signaling, supporting previous epidemiological links between PON2 polymorphisms and diabetic complications. While PON2-deficient mice share several characteristics with PON2-def/apoE−/− mice, such as increased atherosclerotic lesion development, systemic oxidative stress, dyslipidemia, and mitochondrial dysfunction, the latter exhibit improved hepatic insulin signaling compared to apoE−/− controls.
Julia Ebert et al. [65]	2018	C57Bl6/J mice	Insertional mutation in intron 2	8–12 weeks	Standard chow	Coagulation	PON2 deficiency disrupts redox regulation, leading to vascular inflammation and blood coagulation abnormalities. Pon2−/− mice exhibit increased oxidative stress, endothelial dysfunction, elevated IL-6 levels, and heightened tissue factor (TF) activity in endothelial cells. These mice also show shortened coagulation times and increased platelet procoagulant activity. These findings highlight a PON2 redox-dependent mechanism that regulates endothelial TF activity, preventing systemic coagulation activation and inflammation.
Sulaiman D. et al. [66]	2019	C57Bl6/J mice	PON2 deficient	8–10 weeks	Standard chow with ischemia–reperfusion injury induction	Acute myocardial ischemia–reperfusion injury	PON2 protects against acute myocardial ischemia–reperfusion injury (IRI) by mitigating mitochondrial dysfunction and oxidative stress in cardiomyocytes through activation of the PI3K/Akt/GSK-3β RISK pathway.
Diana M Shih et al. [67]	2019	?	PON2 deficient	8–16 weeks	Standard chow for control group and an obesifying diet	Diet-induced obesity	Increased ER stress and mitochondrial dysfunction because of PON2 deficiency lead to decreased energy expenditure, increased adipocyte hypertrophy and obesity in PON2 deficient mice.
Jacqueline M Garrick et al. [68]	2021	C57Bl6/J mice	Insertional mutation in intron 2	3–4 months	Standard chow	Motor behavior	PON2 deficiency induces behavioral changes, particularly affecting locomotion, alongside significant transcript-level biochemical alterations. These changes influence a range of molecular functions linked to affective disorders, cellular differentiation, and cancer biology.
Lisa Spiecker et al. [69]	2021	C57Bl6/J mice	Insertional mutation in intron 2	Adult	Standard chow	effects of inactivation of *PON2* hematopoietic cell differentiation and activity	PON2 plays a key role in regulating HSC functions. The elevated ROS levels in Pon2−/− progenitor cells are associated with an increased frequency of CMPs and GMPs and a shift in the myeloid-to-lymphoid balance in aged mice. Pon2 deficiency activates an anti-apoptotic program in LT-HSCs while also upregulating genes involved in stem cell maintenance, such as Cxcr4, Recql4, and Aatk. We propose that this "maintenance" program compensates for ROS-induced premature aging, ensuring a stable supply of committed progenitor cells in aged mice.
Hagmann et al. [70]	2022	C57Bl6/J mice	Insertional mutation in intron 2	Adult	Standard chow Nephropathy induction with Adriamycin	Diabetic and Inflammatory Glomerular Disease	PON2 knockout mice show heightened glomerular damage under oxidative stress conditions, including adriamycin-induced nephropathy.
Aaron G. Whitt et al. [71]	2023	C57Bl/6/TyrC2J mice	CRISPR/Cas9 single nucleotide insertion in exon 3	Adult	Standard chow	Lewis Lung carcinoma tumors	PON2 was found to be essential for the growth of murine and human lung tumor cells in vitro, but plays a limited role in murine lung tumorigenesis in vivo.
Parameswaran Gangadharan Sreekumar et al. [72]	2023	C57Bl6/J mice	Insertional mutation in intron 2	6–8 weeks	Standard chow + single dose of 20 mg/kg BW NaIO3 administered via the tail vein for retinal pigment epithelial atrophy induction	Age-related macular degeneration	PON2 deficiency leads to mitochondrial dysfunction in retinal pigment epithelial (RPE) cells and a decline in retinal function. These findings suggest that PON2 plays a protective role in retinal health and pathophysiology.
**Author**	**Year**	**Pon3^−/−^ model**	**Modification**	**Age**	**Diet**	**Investigation**	**Conclusion**
Kempster, S. et al. [73]	2011	Sperm from heterozygous Pon3 KO mice were obtained from the Texas A&M Institute for Genomic Medicine	β-geo (lacZ/neo) cassette insertion in first two coding exons	Embryonic and Neonatal	Standard chow	Fetal viability, body and placental weights	PON3 deficiency results in early fetal/neonatal death and reduced fetal growth
Shih, D. et al. [74]	2015	C57BL/6J mice	Neomycin cassette insertion in exon 4	3 months	High-fat “Western” (10 weeks)	Obesity	PON3 KO mice exhibit increased body weight on the high-fat Western diet and altered plasma lipids, increased gallstone weight, enlarged atherosclerotic lesions and higher mortality on the CC diet, indicating that PON3 plays a protective role in metabolic homeostasis
					CC (15.8% fat, 1.25% cholesterol, 0.5% sodium cholate; 16 weeks)	Lipid metabolism, bile acid synthesis, gallstone formation and atherosclerotic lesion development	
Lamichhane, S. et al. [75]	2022	Dahl/SS rat	16 bp frameshift deletion in exon 4 via CRISPR/Cas9	10 weeks	Standard chow (8 weeks) vs. High-salt (8% NaCl; 8 weeks)	Salt-induced renal disease	PON3 KO is linked to significantly increased excretion of cardiotonic steroids, indicating a regulatory role in CTS homeostasis under CKD conditions
Mutchler, S. et al. [76]	2024	B6.129X1-Pon3^tm1Lus/J^	Neomycin cassette insertion in exon 4	Adult	Standard chow vs. High K^+^ (5%) vs. Low K^+^ (<0,003%)	ENaC-mediated Na⁺ reabsorption, renal Na⁺ and K⁺ homeostasis	Loss of PON3 increases ENaC activity, leading to lower plasma K⁺ and enhanced NCC phosphorylation; these changes are normalized by a high K⁺ diet, underscoring PON3’s critical role in renal electrolyte balance and Na⁺ transport regulation

## Data Availability

No new data were created or analyzed in this study. Data sharing is not applicable to this article.
